# A Question Never Comes Alone: Comments on ‘What is Aging?’

**DOI:** 10.3389/fgene.2012.00150

**Published:** 2012-08-23

**Authors:** Margarida Matos

**Affiliations:** ^1^Centro de Biologia Ambiental, Departamento de Biologia Animal, Faculdade de Ciências, Universidade de LisboaLisboa, Portugal

One of the neglected issues in this article is the role of genetic variation with age-independent genetic effects. Genetic variation affecting aging and other life-history characters can, in theory, be maintained by mutation-selection balance for genes that are age-specific in expression, where the expectation is a higher additive genetic variance of fitness components at more advanced ages. However, this mechanism – accumulation of mutations – though leading to the maintenance of genetic variation, does not involve a consistent performance across ages, and thus does not produce a plateau late in life characterized by the type of positive correlations assumed by lifelong heterogeneity theories. But in any case, both age-independent and age-dependent mechanisms will play a role in defining the timing and state at which aging stops.

## Given the Present Knowledge that Aging May Stop at an Advanced Age, Do We Require a Re-Definition of Aging?

Before going into that “new” definition, we need to distinguish between: proximal and ultimate causality; thus aging may be due to a deterioration of physiological functions as *proximate* cause, and due to a decline of the forces of natural selection as *ultimate* cause. This decoupling leads to different levels of understanding and predictions; e.g., only the latter leads to both the prediction that aging is multifactorial and *also* that it can stop at advanced ages. Moreover, even considering just the first level of understanding, a common confusion occurs between correlation and causality. This can even lead to such claims as that aging is due to a reduction of the size of telomeres with age, a clear confusion between association and causality, with no power to add understanding to the deep, general, evolutionary causes of aging.

In light of this, there is a potential problem with the definition of what is “internal physiological deterioration,” used in the “Evolutionary Biology of aging” book (Rose, 1991). In fact, the term may be interpreted wrongly as if “some” physiological processes can be disentangled from the environments with which they interact. All processes do in fact depend on interactions between genotypes and the environment, with the latter being both “internal” and “external” factors. As an illustration, once a population starts aging, the subsequent unfolding of aging processes will be affected by the cumulative effects of deterioration also, both by the “external” and “internal” environment (since natural selection will decline even faster when mortality at late ages rises, due to both “internal” and “external” deterioration of functions with age, in an exponential way). The evolution of aging is thus an integrative process, involving all kinds of factors, and the statement “internal physiological deterioration” may convey a wrong message. In other words, speaking about “internal” processes may give a wrong idea that *before* aging evolves there is already the potential for the deterioration of particular physiological mechanisms. A better definition, taking this into account, as well as the fact that the *ultimate causes* of aging lead to a prediction that from a certain point on aging stops, might be the decline of *age-dependent* physiological functions. This leaves aside physiological functions (whichever they may be) that affect the performance across all life (i.e., with positive correlation across ages), as well as factors such as damage, diseases, etc., inflicting the same degree of deterioration independent of age.

## How Much Can We Shape the Change of Performance with Age?

At a first glance, it is intuitive that an environment with “benign conditions” leads to a lowering of age-independent mortality, rendering more likely that a plateau is expressed, as it increases the probability that an individual survives past the age where aging stops (the “breakday”) and thus enters the “plateau phase.” But the classification of an environment as “benign” or “detrimental” depends on the evolutionary history of the population: e.g., can new environments, even if “stress free” be considered benign? Can environments where the population has been long adapting, even if “stressful,” be considered “detrimental”? This is not superficial rhetoric, since we have seen recurrent arguments in the literature that defend contrasting expectations for the evolution of aging, relative to the general theory of aging, as a function of the environment/history of the populations. This was the case with the old “Giesel” argument that a stressful long-term laboratory environment leads to an evolutionary trade-off between early and late performance in terms of fitness, that is not the “true” pattern under mild conditions (Giesel et al., 1982; but see Rose, 1984). It has also been invoked more recently by several researchers (e.g., Promislow and Tatar, 1998; Sgrò and Partridge, 2000; Hoffmann et al., 2001; Linnen et al., 2001) who defend the idea that a “relaxed” environment such as the lab leads to the accumulation of mutations that inflate the aging pattern, arguing again that the lab is not a “real” scenario, and that the evolution of aging (or related traits, such as resistance to a stress) should be analyzed under more “natural” conditions. What is correct is to consider the differences between populations under different age-dependent selection regimes in an environment where populations have already adapted, whatever the specificities of that environment. Given this, we may say that by “benign” is meant an environment where populations have had the opportunity to adapt.

An illustration may be changes in nutrients: does an increase in the concentration of yeast improve a *Drosophila* environment? Not necessarily: it may lead to higher reproduction at the expense of survival, and thus to a quicker aging while the populations are still adapting to these new conditions. This in turn will render it less likely that an individual survives past the “decline” (aging) phase, reaching the phase where aging stops (the plateau). But given enough time, maybe the populations do increase their capacity to assimilate the new concentration of nutrients, improving all fitness components, and thus reducing the aging rate and increasing the chances of reaching a lower plateau (in terms of mortality). On the other hand, it is possible that a constraint such as lower nutrients leads to a reduction of early fecundity and an improvement in longevity, again far from equilibrium, affecting the probability that an individual reaches the age where the plateau is expressed. Again, only after evolution in that environment can we see whether or not such a change allows a higher likelihood that a plateau is expressed.

But the question remains: can we manipulate the environment in order to render it more likely that an individual reaches the plateau? Unfortunately there is no simple “recipe” at present. The important message is that we cannot neglect the history of a population, and a key factor may be considering the ancestral environment where populations have evolved, particularly for populations that are at present under novel conditions. For example, in humans recent drastic changes of life-style may have led to faster senescence and a rise in the value and age where a plateau may be expressed.

## One Important Issue is How Independent is the “Breakday” from the Rate of Senescence and the Plateau Value. Can We Disentangle the Three?

It is fairly intuitive that populations that have a slower senescence rate will present at advanced ages a lower plateau than populations that age faster. But, as the plateau derives from the evolution “after” selection ends, i.e., at ages under the “selection shadow,” it is expected that a quicker decline of selection with age will lead to a more “precocious” age when the plateau is reached. In other words, faster senescence is associated with a younger age where the plateau is expressed (smaller break day). The question is: can we disentangle the three parameters: rate of senescence, level of plateau, and age at which it is reached? It is interesting to suppose that we could by genetic changes or environmental manipulation slow aging in a population so that a 50-year-old human could be as vigorous as a 30-year old in the unmanipulated group; but would not it be better if we could “manipulate” aging so that aging stopped at 30? Is this possible, in genetic and environmental terms?

## Can Aging Be Reversed at the Individual Level?

If aging is not a progressive deterioration with age, could the effects of aging be reversible? Could we target the physiological functions that have a genetic basis that is age-specific, or that have a genetic trade-off across ages – which cause aging – and which have a universally positive effect across ages – which are the ones responsible for the plateau? In fact, there is empirical evidence that late-life physiology is distinct from the physiology of aging (Shahrestani et al., 2012). As there is redundancy of functions in our genome, could it be possible to compensate for the loss of functions of those genetic mechanisms which cause aging by activating the over-expression of other genes that confer stable performance throughout life?

Furthermore, we need to distinguish two levels of definition of a phenomenon, as *state* and as *process*; taking the example of the concept of adaptation. It is fundamental to be clear whether we consider the concept as capacity to respond to the environment – as *state*, and, in the latter case, whether directly or as a by-product of selection – and as the *process* of becoming adapted, that is, natural selection *per se*. This is not an irrelevant issue, as it is at the core of critics such as the old argument of Popper in the 1970s that natural selection is a tautological theory. The “solution” came from Dunbar (1982), as well as Sober (1984), with the proposition that a distinction between adaptation – as state – and fitness is essential to uncouple the two, rendering the outcomes of selection not inevitably as a rise in adaptation (=fitness). Taking this difference into account, we see how it may affect our perception of such complex phenomena as aging. In particular, there is evidently a substantive difference between considering aging as “ultimate process” or as “immediate state.” Such different perspectives and limitations, as well as the important distinction between correlation and causality (see above), are essential for progress on the connection between evolutionary and genetic causes of aging, particularly how environmental and/or genetic manipulation may affect the physiological changes that occur with age.

Concerning commentary of T. F.: I think clarification is needed concerning the common features of previous definitions of aging versus those definitions that clearly state that aging can eventually stop at advanced ages. In this sense, I have to disagree with T. F., both with his statement about what Rose's present definition of aging is (a decline or loss of adaptation with increasing age, caused by a time-progressive decline of Hamilton's forces of natural selection) and about T. F.’s discrimination between *progressive* and *persistent* decline. I think that M. R. R.’s phrase *aging might be best conceived as a facet of adaptation, specifically its de-tuning during the first part of adulthood* illustrates that a new definition should incorporate the idea that aging is a *phase* which occurs during the first part of adulthood, and not a process that will go on till death. In that sense my modest proposal, which is nothing fundamentally new but may be useful for its simplicity and for forestalling misleading conclusions, is that aging is a decline of *age-dependent* physiological functions (see below).

Concerning commentary of J. L. G.: I confess that I strongly mistrust results of genetic analysis involving mutant strains when addressing the evolutionary genetics of sexual random mating populations, as such analysis may inflate the effect of mutations that may be irrelevant for the evolution of outbred populations. Having said this, I think that such new techniques as NGS may allow us to tackle the most relevant issue of how genes with age-dependent versus age-independent affect fitness-related traits. My bet is that the data obtained by Chintapalli et al. (2007) cited by J. L. G. are substantial over-estimates of the number of genes that have effects which are independent of age. Though it seems to me that the simple observation of the patterns of aging imply such results, only future research can tell whether this conjecture is right or wrong.

Concerning commentary of D. E. M.: I agree that it seems counterintuitive that such a general explanation for aging – the decline of the forces of natural selection with age – allows some organisms to live for very long time without clear signs of senescence, or even at least apparently not aging. While for some time one simple explanation was that populations where there is no separation between soma and germline do not senesce – and this is clearly not a contradiction but a corollary of the Hamiltonian theory of aging – it is now becoming frequent to find that aging is a much more universal phenomenon. In fact, bacteria, which were a model example of the absence of aging, do age, and they also “obey” the general condition that they have a distinction between the “mother” and “daughter” line, in other words of germ and soma (though not in separate tissues, obviously, as this is not required, as has already been seen in yeasts). As it is always easier to prove that a phenomenon occurs than that it does not, the apparent mystery of the absence of aging in some organisms may be just a result of a diversity of rates of aging due to different age-independent mortalities, mortality patterns that in turn lead to very slow declines in the forces of natural selection, and thus of aging, in the paradigmatic organisms that exhibit very long lifespans.
